# Gene copy number variation throughout the *Plasmodium falciparum *genome

**DOI:** 10.1186/1471-2164-10-353

**Published:** 2009-08-04

**Authors:** Ian H Cheeseman, Natalia Gomez-Escobar, Celine K Carret, Alasdair Ivens, Lindsay B Stewart, Kevin KA Tetteh, David J Conway

**Affiliations:** 1 Department of Infectious & Tropical Diseases, London School of Hygiene & Tropical Medicine, Keppel Street, London, WC1E 7HT, UK; 2 Medical Research Council Laboratories, Fajara, PO Box 273, Banjul, The Gambia; 3 Wellcome Trust Sanger Institute, Hinxton, Cambridgeshire, UK

## Abstract

**Background:**

Gene copy number variation (CNV) is responsible for several important phenotypes of the malaria parasite *Plasmodium falciparum*, including drug resistance, loss of infected erythrocyte cytoadherence and alteration of receptor usage for erythrocyte invasion. Despite the known effects of CNV, little is known about its extent throughout the genome.

**Results:**

We performed a whole-genome survey of CNV genes in *P. falciparum *using comparative genome hybridisation of a diverse set of 16 laboratory culture-adapted isolates to a custom designed high density Affymetrix GeneChip array. Overall, 186 genes showed hybridisation signals consistent with deletion or amplification in one or more isolate. There is a strong association of CNV with gene length, genomic location, and low orthology to genes in other *Plasmodium *species. Sub-telomeric regions of all chromosomes are strongly associated with CNV genes independent from members of previously described multigene families. However, ~40% of CNV genes were located in more central regions of the chromosomes. Among the previously undescribed CNV genes, several that are of potential phenotypic relevance are identified.

**Conclusion:**

CNV represents a major form of genetic variation within the *P. falciparum *genome; the distribution of gene features indicates the involvement of highly non-random mutational and selective processes. Additional studies should be directed at examining CNV in natural parasite populations to extend conclusions to clinical settings.

## Background

Many classes of genome sequence variation occur in the malaria parasite *P. falciparum *that may contribute to variation in pathogenicity, including single nucleotide polymorphism (SNP) [1], insertion and deletion of short sequences (indels) [2], large scale deletions [3], amplifications [4], inversions [5], and translocations [6]. Analysis of diversity in the ~22.8 Mb parasite genome has focused predominantly upon the detection of SNPs and indels [7-9] by DNA re-sequencing methods, although the power of oligonucleotide microarray hybridisation for detecting sequence polymorphism has also been applied [10,11]. This has shown that *P. falciparum *genes encoding surface proteins possess a higher than average level of sequence diversity, whereas genes encoding proteins with mitochondrial, metabolic and cell growth functions have low diversity [8,9]. Recombination rate has also been shown to be variable along the length of each chromosome, being particularly high in the sub-telomeres [8,12].

Genome sequencing can be utilised for detecting gene deletions and amplifications [13,14] although alternative methods such as DNA microarray platforms also allow copy number variable regions of the genome to be robustly detected on a large scale. These CNV regions play a prominent role in the adaptive biology of the parasite. For example, sub-telomeric deletions on chromosomes 2 and 9 have been linked to a loss of infected erythrocyte cytoadherence through deletion of genes encoding adhesion molecules [3,15]. Conversely, mefloquine resistance is conferred through amplification of a multi-genic locus on chromosome 5 including the multi-drug transporter *pfmdr1 *gene [4]. Hybridisation of labelled genomic DNA to microarrays can detect amplified or deleted sequences, and depending upon probe density the spectrum of DNA sequence variation that can be detected ranges from SNPs up to copy number variants in the Mb size range [16,17]. Early studies using microarray platforms [18,19] demonstrated polymorphism within the sub-telomeres of chromosomes in the Hb3 strain and a panel of sub-clones from a single *P. falciparum *isolate (IT/FCR-3) respectively, and more recent studies identified novel CNV genes by using arrays to assay small panels of different *P. falciparum *isolates [10,11,20].

CNVs have been shown to be correlated with repetitive sequences in the genomes of humans and model organisms, highlighting the role of non-allelic homologous recombination in the genesis of variants [21-24]. Consistent with this, amplicon breakpoints in the *P. falciparum *mefloquine resistance locus on chromosome 5 and surrounding the GTP cyclohydrolase amplicon are within microsatellite regions or repetitive monomeric A/T tracts [25,26]. In comparison with SNP mutation rates in eukaryotic genomes in the order of ~10^-8^-10^-9 ^per nucleotide site per generation, it has been estimated that large deletion or amplification mutations occur at a rate ~10^-4 ^per nucleotide site per generation [22,27]. It has been postulated that gene duplications safeguard against deleterious mutations [28], and allow more rapid rates of evolution within coding sequences [29], as one gene copy retains gene function whilst the other has reduced constraint to accrue new mutations. Conversely, deletions are suggested to be more often under purifying selection, and thus purged from populations [30].

To identify commonly amplified and deleted genes, and test for associations with factors that could contribute to their generation and selection, a genome-wide survey of CNV genes was performed on diverse cultured lines of *P. falciparum*, using a high-density oligonucleotide microarray platform.

## Results

### CNV genes are non-randomly distributed throughout the *P. falciparum *genome

**Figure 1:**
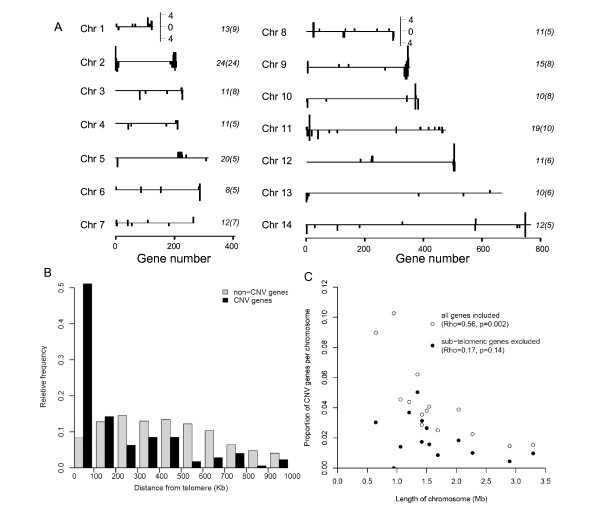
**The genomic location of CNVs**. (A) Frequencies of CNV for each gene along the genome for each chromosome is shown above the axis for amplifications and below the axis for deletions. Total numbers of CNV genes are given at the left hand side of each chromosome with the number of CNV *var*, *rifin *and *stevor *genes in brackets. (B) Distance to telomeric ends is shown to be significantly decreased in CNV genes (grey bars) in comparison to non-CNV genes (hashed bars). (C) Fraction of a chromosomal gene content classed as a CNV gene declined as number of genes per chromosome increased (open circles), this trend became weaker when the chromosomal portion excluding the sub-telomeric ends was examined (black closed circles). A significant divergence in gradient from 0 was detected only when all genes were analysed together, with multiple-adjusted r^2 ^values indicating a prominent role for chromosome length in predicting its proportion of CNV genes.

Genomic DNA extracted from 16 *in vitro *cultured isolates was hybridised to the high density custom Affymetrix oligonucleotide microarray PFSANGER. CNV genes were identified as those possessing a log_2 _ratio in any isolate that was in excess of 1 (gain) or below -1 (loss) corresponding to a doubling or halving of signal intensity compared to the median signal intensity for that locus across all isolates (see Materials and Methods). In total, 186 genes showed hybridisation signals consistent with deletion or amplification in one or more isolate. In each isolate between 11 and 37 genes showed evidence of deletion or amplification (see Additional file 1). These putative CNV genes were observed on all 14 chromosomes, though the pattern of variation was non-random (Figure 1A). There was an excess of CNV genes towards chromosomal ends, with 113 (60.8%) CNV genes within previously defined chromosome sub-telomere regions (n = 625) [31,32], and 73 (39.3%) at more internal chromosomal loci (n = 4683, Figure 1A,B). Hence, 18.1% of genes in the subtelomeres are observed as CNV compared with 1.6% in internal regions. This was exemplified by chromosome 2, on which all CNV genes were detected in sub-telomeric regions (Figure 1A and Additional file 2). Overall, there was a highly significant skew toward sub-telomeric location for CNV genes compared with non-CNV genes (*p *< 0.0001, Kolmogorov-Smirnov test; Figure 1B, Table 1). The proportion of all genes on each chromosome detected as CNV genes decreased with chromosomal length, as expected since sub-telomeric regions take up a lower proportion of the larger chromosomes (Figure 1C).

**Table 1:** Properties of CNV genes compared with non-CNV genes in *P. falciparum*

Property		Common CNVs (n = 87)			Rare CNVs (n = 99)		
		*p*-value	Observed	Expected	*p*-value	observed	expected
Proximity to centromeres^b^	↑	**0.012**	n/a	n/a	**0.0001**	n/a	n/a
Proximity to telomeres^b^	↑	**<0.0001**	n/a	n/a	**<0.0001**	n/a	n/a
Signal peptide^a^	n/a	0.92	14	13.8	0.59	18	13.1
Tm domain^a^	↑	**0.027**	35	25.24	**0.0002**	48	30.35
Length (bp)^b^	↓	**<0.0001**	n/a	n/a	**<0.0001**	n/a	n/a
Proximity to segmental duplications	↑	**<0.0001**	n/a	n/a	**<0.0001**	n/a	n/a
Rodent malaria parasite orthologue^a^	↓	**<0.0001**	13	64.38	**<0.0001**	32	77.42
*P. knowlesi *orthologue^a^	↓	**<0.0001**	15	64.5	**<0.0001**	37	77.56
any orthologues^a^	↓	**<0.0001**	18	55.3	**<0.0001**	37	66.5

Expected values were calculated under the assumption that each property is distributed evenly throughout the genome

Values where *p < 0.05 *shaded in bold

^a^Chi-squared test; ^b^Kolmogorov-Smirnov test

The sub-telomeres are typified by repeat regions, large multi-gene families (*var, rifin, stevor*) and segmental duplications and share considerable homology among chromosomes. There was no association of CNV genes with internal *var *clusters on chromosomes 4, 6, 7, 8 and 12, nor with identified segmental duplications, and only a weak association with centromeres was shown (Table 1). A strong association (*p *< 0.0001) with proximity to segmental duplications was however observed.

### Detection of previously identified CNV genes

**Figure 2:**
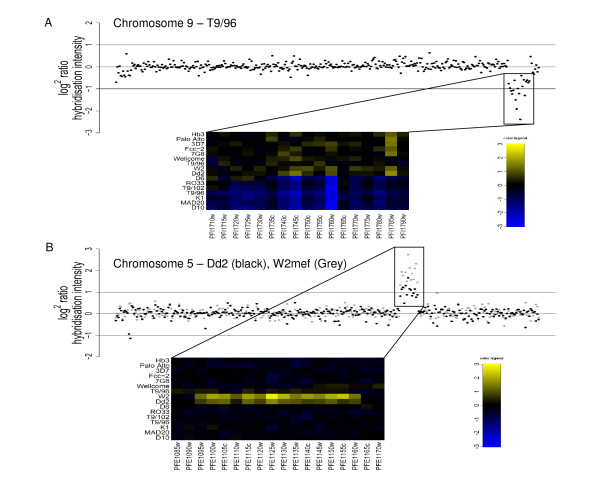
**CNV regions in the *P. falciparum *genome**. Detection of previously described multi-genic deletions and amplifications. Plots show log_2 _hybridisation signal of individual genes in physical order along chromosomal length with cut-offs for amplification/deletion of a gene shown by lines at 1/-1 respectively. Heatmaps show CNV regions at greater resolution for all hybridised strains with genes amplified or deleted as indicated by the colour key (yellow and blue respectively). A. Multi-genic deletion on chromosome 9. B. Multi-genic amplification on chromosome 5.

Several phenotypically important CNV regions have been previously characterised in the parasite lines investigated in this study. These include a sub-telomeric deletion of a region of chromosome 9 including the annotated genes cytoadherence linked asexual gene 9 (*clag9*, PFI1730w), ring exported protein (REX, PFI1735c) and gametocyte implicated protein (PFI1720w). This truncation is frequently observed during adaptation to long-term culture in *P. falciparum *strains [15,33,34]. This was seen in D6, RO33, T9/102, T9/96, K1, MAD20, and D10 in our experiments, extending beyond *clag9 *in all strains except D6 where hybridisation indicates a deletion may have occurred between *clag9 *and PFI1735c (gene for the ring exported protein, REX, Figure 2A).

Mefloquine resistance is caused by amplification of a multi-genic locus surrounding the *pfmdr1 *gene on chromosome 5 (PFE1150w) and has been shown to be highly copy number dependant [4]. The Dd2 and W2mef parasites are related clones, derived from a single progenitor selectively grown under mefloquine selection to produce drug resistant parasites [35]. Here, hybridisation indicated an increased signal for *pfmdr1 *in Dd2 and W2mef (2.2 fold and 3.5 fold increase in signal intensity respectively, Figure 2B). The extent of the amplification in Dd2 encompassed 14 genes from PFE1095w to PFE1160w, in complete agreement with previously published microarray data [20]. Here the exact same boundaries of the amplification are found for W2mef.

### CNV genes are mostly rare and non-consecutive

**Figure 3:**
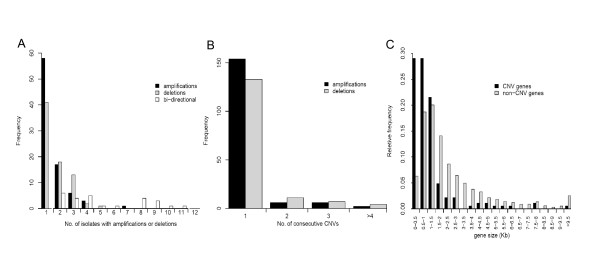
**Features of CNV dataset**. (A) 99 CNV genes (58 amplifications, 41 deletions) were detected in a single isolate whereas 87 were replicated between isolates and classed as common CNVs. (B) Consecutively detected CNV genes are infrequent for both amplifications (black bars) and deletions (white bars), the vast majority being non-adjacent to variable regions, the few large variants that we detected have been previously described and include sub-telomeric truncations in chromosomes 2 and 9 and amplification of the *pfmdr1 *gene locus on chromosome 5. Within consecutive CNV regions it was rare to find hybridisation signals uniformly outside of the cut-offs. Genes that precluded detection using this stringent cut-off though showed clear patterns of variation were identified as those adjacent to identified CNV genes and possessed a log_2 _ratio of +/- 0.8. (C) Length of CNV genes was shown to be a highly significant property of CNV genes being smaller than non-CNV genes.

Approximately half of the total of putative CNVs (99/186, 53%) were each detected in only a single parasite line (Figure 3A). A slight bias in these rare CNV genes was noted, amplifications (n = 58) being more frequent than deletions (n = 41) compared with 27 amplifications and 34 deletions detected in 2 or more isolates (*p *= 0.055, Fisher's exact test). Further analysis focused upon the 'common' CNV genes, detected in 2 or more isolates, as these were considered most important and least likely to have originated in culture or to be due to any artefact (Figure 3A), this still represents a substantial level of genetic variation affecting 0.7% of the coding sequence of the genome.

The distribution of size polymorphism in the *P. falciparum *genome and the number of consecutively identified CNV genes was determined, revealing relatively few runs of two or more CNV genes (Figure 3B). There was also a trend towards small genes (<1 kb) being more frequently variable than larger genes (*p *< 0.0001, Kolmogorov-Smirnov test; Table 1, Figure 3C).

### Confirmation and identity of deleted genes

Validation of deletions in the Dd2 and Hb3 genomes was performed using bioinformatic searches of published sequence data aligned to the 3D7 genome. Eleven of 14 putative deletions in Dd2 from the microarray data were supported by the absence of the gene in the Dd2 genome sequence (see Additional file 3), the exceptions being PF14_0040 and PF11_0061 (containing polymorphisms overlapping 27% and 33% of probe target sequences respectively), and PFE0070w (showing extensive re-arrangements along its length due to interspersed repeats). Five of 7 putative deletions in the Hb3 genome were confirmed by the absence of the genes in the Hb3 sequence (see Additional file 3), the exceptions being PFF0860c (containing differences in the Hb3 sequence that overlapped 44% of probe target sequences) and PFI0060c (in which all probes were affected by sequence polymorphism). As a control comparison, a random set of 500 genes (~10% of the genome) that were classed as 'present' in both the Dd2 and Hb3 hybridisations were examined. 97.8% of these genes were identified in the Hb3 sequence suggesting that deletions confirmed by bioinformatic methods are unlikely to be a product of lack of genome coverage, whereas a smaller majority (76.6%) was identified in the Dd2 sequence, consistent with the higher number of unclosed gaps in the current draft Dd2 genome compared to Hb3.

This high level of confirmation of results from the array platform allowed the identification of novel deletions to be catalogued in the *P. falciparum *genome. For example, the *pfsbp1 *(PFE0065w) gene was determined as 'deleted' along with an adjacent gene (*interspersed repeat antigen*, PFE0070w) in three strains (Dd2, Fcc-2 and 7G8). Examination of the Dd2 genome sequence revealed a truncation of the *pfsbp1 *gene (contains only the first 444 bp, out of 1012 bp in 3D7) explaining the low hybridisation signal observed on the microarray. Gene knock-out studies on *pfsbp1 *(PFE0065w) encoding the skeleton binding protein, have previously shown it as important in the trafficking of PfEMP1 to the erythrocyte cell surface [36,37]. Deletion of two glycophorin binding protein genes with high (>93%) sequence identity on different chromosomes was also identified: *gbph *(PF14_0010) in Dd2, Fcc-2 and 7G8 and *gbph2 *(PF13_0010) in D10, Fcc-2 and T9/102. As deletion of both genes was detected in a single strain (Fcc-2) it is likely that function is either complemented by gene paralogues (i.e. PF10_0159, the glycophorin-binding protein 130 precursor) or redundant for *in vitro *cultured parasites.

Overall, the putative CNV genes included many hypothetical genes (n = 96/3371, 2.9% of all hypothetical genes). The proportion of CNVs was elevated in the *rifin *(n = 20/180, 11%) and *stevor *(n = 3/12, 25%) gene families. Other gene families also showed a high proportion of CNV genes, *pfmc-2tm *(n = 6/12, 50%), large ribosomal subunit protein (n = 5/48, 10.4%), histone (including variant and putative histone proteins) (n = 3/8, 38%), FIKK protein kinase (n = 2/20, 10%) and *etramp *(n = 2/11, 18%, see Additional file 1). However, a high proportion of CNV was not detected among *var *genes (n = 1/59, 1.8%), though truncated and pseudogenic *var *genes exhibited high levels of CNV (n = 5/22, 22.7%; 8 genes had low or no probe coverage), confirming that the polymorphism observed in PfEMP1 proteins is most probably due to recombination events between members of the *var *genes rather than amplification and/or deletion [38,39].

### CNV genes share common features

There was a deficit in CNV genes with orthologues to other *Plasmodium *species (Table 1) with 67.2% of common CNVs and 56.2% of rare CNV genes being *P. falciparum *specific. A large number of these species-specific genes in the *P. falciparum *genome are exported to the infected erythrocyte cell surface and possess transmembrane domains, though we found no excess of CNV among genes containing the major signal peptide associated with export to the cell surface (the PEXEL motif), and only a weakly significant excess of genes containing transmembrane domains (Table 1).

Duplicated genes have been hypothesised to possess a higher than neutral level of polymorphism due to relaxation of selective constraint. We used a published dataset of 3,539 values [8] of per gene nucleotide diversity (π) to determine if the CNVs detected in this study showed increased sequence polymorphism, compared to non-CNV genes. A significant excess of polymorphism was detected for both the distribution of π values (*p *= 0.018, Kolmogorov-Smirnov test), and the proportion of CNV genes with values of π greater than the overall average (4.83 × 10^-4^, *p *= 0.011, chi squared test, Pearson corrected). There was a slight excess of deleted genes, with 57% of CNV genes above the average pairwise π being deleted and 43% being amplified, although this was not significant (*p *= 0.07, two-tailed, Fisher's exact test, see Additional file 4).

No association was seen in the distribution of monomeric A/T tracts in CNV genes and 1 kb of surrounding intergenic sequence up- and down-stream of the gene coding sequences, compared to non-CNV genes (*p *= 0.08, Kolmogorov-Smirnov test, see Additional file 5). This was consistent when CNV in subtelomeric genes or non-subtelomeric genes were considered separately (non-CNV vs. CNV in sub-telomeric regions, *p *= 0.10; non-CNV vs. CNV in non-subtelomeric regions *p *= 0.98, Kolmogorov-Smirnov test). However, sequence encompassing amplified genes (n = 80) showed a significantly skewed distribution toward shorter A/T tract lengths when compared to deleted genes (n = 71, *p *= 4.6 × 10^-6^, Kolmogorov-Smirnov test) and non-CNV genes (n = 5176, *p *= 1.3 × 10^-5^, Kolmogorov-Smirnov test).

SNPs located within probe sequences have been shown to reduce binding of target DNA hybridised to the array, in the case of exceptionally highly polymorphic genes. To determine if the modest excess in polymorphism observed in deleted CNVs was due to an artefact of lower hybridisation, the relationship between sequence diversity and hybridisation signal was examined. SNPs relative to the 3D7 genome were downloaded from whole genome sequencing projects for the parasite clones Dd2 and Hb3 http://www.PlasmoDB.org. Overall, no association between the gene level hybridisation signal and the density of SNPs in each gene (compared to 3D7) was observed (see Additional file 6 and 7).

## Discussion

The distribution of CNV genes in *Plasmodium falciparum *shows consistency with what has been seen in the genomes of higher eukaryotes [29,30]. The excess of smaller sized CNV genes is in accordance with general predictions [30,40]. The small proportion of consecutive CNV genes indicates that CNV regions in *P. falciparum *are mostly very small, although there are notable exceptions previously known that were also detected here. Sub-telomeric regions of *P. falciparum *have evolved as hotspots of genetic diversity, typified by a high proportion of species specific genes, segmental duplications, nucleotide diversity, high mutation and recombination rates. Results here show that CNV genes are also common in sub-telomeric regions, and have highlighted features relevant to the mutational origin and selective pressures of CNV genes in *P. falciparum*. Given the pivotal role of segmental duplications in CNV generation in the genomes of humans and mice [21,22], it seems likely that a similar role could occur in malaria parasites. No complete segmental duplications were detected as either amplified or deleted in this study, though it has been previously shown that segmental duplications are copy number variable in the *P. falciparum *genome resulting in transcriptional up- and down-regulation of their gene complement [20,31]. The sub-telomeres possess large gene families containing regions of high sequence homology, and we have identified a number of these as being putative CNV genes. This suggests that mechanisms such as non-allelic homologous recombination (NAHR) may be key to the generation of copy number variation, through recombination of regions of homology either intra- or inter chromosomally [27].

The function of deleted genes may be frequently complemented by closely related genes, suggesting that most CNV has no adverse effect on phenotype, and is thus nearly neutral [21]. However, *in vitro *culture of parasites might allow the maintenance of a greater number of mildly deleterious alleles than in natural populations due either to the reduction in effective population size that would generally increase the effect of genetic drift, or to the relaxation of some specific selective constraints *in vitro*. This is highlighted by the deletion *in vitro *of *pfsbp1*, an important transporter of PfEMP1 to the erythrocyte cell surface, in three of the parasite lines, a loss that may be non-viable in natural infection due to a failure to cytoadhere to the microvasculature and evade immune responses. An unknown proportion of the variation presented here may not have originated in the natural environment, so parasites sampled directly from patients will need to be examined to exclude the effect of culture adaptation.

Genomic organisation between rodent malaria parasites and *P. falciparum *is well conserved, and arranged in large blocks of syntenic genes, between which species specific genes have evolved [41]. The deficit of CNV genes with orthologues to rodent or macaque malaria parasites suggests two possible causes that are not mutually exclusive: 1) there is strong negative (purifying) selection on the "core" *Plasmodium *genes and disruption of these has a deleterious effect. 2) *P. falciparum *specific genes tend to be under strong positive (diversifying) selection and this favours the maintenance of population-wide CNV.

Many of the *P. falciparum *specific genes encode known antigens and exported proteins from the intra-erythrocytic stage of the parasite lifecycle. The presence of multiple copies of these genes allows the generation of antigenic variants within a single genome, greatly facilitating immune escape of the parasite. A significant association with increased nucleotide diversity in the CNV genes identified in this study is consistent with this. The higher values of π detected in loss CNVs may be attributable to technical limitations of the comparison, there was poor sequence coverage of CNV genes in the dataset used (29% of CNV genes with values for π, compared to 67% of all genes in the genome) [8]. High coverage sequencing that is underway on a large number of *P. falciparum *isolates will further refine our understanding of evolutionary constraints and modifications on the genome.

## Conclusion

Our results demonstrate the existence of gene amplifications and deletions (copy number variations, CNVs) throughout the *Plasmodium falciparum *genome by comparing diverse culture-adapted isolates. These gene CNVs correlate to subtelomeric location, short gene length, nucleotide diversity and low orthology to closely related *Plasmodium *species. We have discussed CNV as an important evolutionary mechanism, allowing the adaptive expansion of diverse gene families whilst copy number of core "housekeeping" genes are maintained by negative selection. In addition to this we have noted the occurrence of previously undetected gene CNVs and highlighted those with a potential phenotypic importance to clinical disease. However, host selective pressures are relaxed *in vitro *and may allow for some deleterious rearrangements than would not be seen *in vivo*, so studies directly on clinical isolates will also be important.

## Methods

### *P. falciparum *culture, gDNA preparation and strain identification

*P. falciparum *culture-adapted parasite lines were obtained either from the MR4 repository or were kindly provided by Prof David Walliker, Prof Richard Carter (University of Edinburgh) or Dr Livia Vivas (LSHTM). Parasite culture in erythrocytes was performed using standard methods [42], and genomic DNA (gDNA) was extracted using the QIAquick whole blood extraction kit (QIAGEN) and eluted into 30 μl of a low salt buffer.

Genomic DNA from each cultured isolate was first examined by sequencing the highly polymorphic *block 2 *region of the *msp1 *gene as a check for identity. Two pairs of cultured lines (MAD20/D10 and T9/94/Wellcome) had identical sequences at *msp1*, and subsequent sequencing showed each pair was identical at 24 other polymorphic genes. It was thus considered that these identical pairs were the product of cross-contamination, though the exact timing of this was unknown as all isolates were cultured independently in our study. The panel of parasite lines nevertheless represents a geographically diverse set of isolates, with 3 nominally from Africa (D6, RO33, Palo Alto; apart from 'Wellcome' that was originally supposedly African but was identical to T9/94 here), 7 from South East Asia (T9/96, T9/102, K1, Fcc-2, T9/94, and Dd2 and W2mef that are almost identical clones from the same isolate), 2 from Papua New Guinea (MAD20 and D10 with apparently shared clonal history in culture), one isolate from South America (7G8), one from Central America (HB3), and one (3D7) which was cloned from isolate NF54 derived from a case of airport malaria in The Netherlands presumed to be acquired from the bite of an infected African mosquito.

### Preparation and hybridization to Affymetrix PFSANGER microarray

12 μg genomic DNA from each of the 16 cultured lines was fractionated by digestion with A*poI *for 2 h at 50°C, followed by incubation for 20 min at 80°C. Digested DNA was then extracted by addition of phenol-chloroform-isoamyl alcohol volume/volume, and precipitated in 0.1 × volume 3 M sodium acetate at pH 5.2 and 2 × volume 100% ethanol. Pelleted DNA was washed in 70% ethanol then air dried and re-suspended in 20 μl de-ionised H_2_O. End labelling of the DNA was then performed in terminal deoxynucleotidyl transferase buffer and 5 mM CoCl_2 _(Roche) by addition of 1 nM biotin-N11-ddATP (Molecular Probes) and 400 units terminal deoxynucleotidyl transferase (Roche) for an hour at 37°C in preparation for hybridisation.

The Affymetrix PFSANGER GeneChip is a tiling-like array, with oligonucleotide probes that can overlap across unique sequence regions of the genome [11,43]. It is composed of 2,439,646 perfect match-only probes (PM) designed using the January 2005 sequence data of the 3D7 *P. falciparum *strain as a reference. 1,219,157 of the probes map to predicted gene coding sequences with representation of most of all annotated and hypothetical genes. Hybridization was performed after equilibration of the array at room temperature and incubation with pre-hybridization buffer in an Affymetrix oven at 45°C for 10 min rotating at 60 rpm. A mixture containing the labelled genomic DNA of a particular *P. falciparum *isolate, eukaryotic hybridization controls (Affymetrix), oligoB2 control (Affymetrix), Herring sperm DNA (Promega), acetylated BSA (Invitrogen), in pre-hybridization buffer was incubated at 99°C for 5 min, and then at 45°C for 5 min. This mixture was then used for hybridisation at 45°C for 16 h under constant rotation at 60 rpm.

After hybridization, the arrays were washed and stained in an Affymetrix wash station following Affymetrix Eukaryotic protocol, in a series of washes in non-stringent buffer (6× SSPE, 0.01% Tween-20) followed by stringent washes (100 mM MES, 0.1 M NaCl, 0.01% Tween-20). The GeneChips were stained by addition of 10 μg ml^-1 ^R-phycoerythrin streptavidin in staining buffer (100 mM MES, 1 M NaCl, 0.05% Tween-20) with 2 mg ml^-1 ^acetylated BSA for 5 min at 35°C then washed in non-stringent buffer. Amplification of the staining signal was performed by addition of 3 μg ml^-1 ^biotinylated antistreptavidin antibody (Vector Laboratories) in staining buffer, 2 mg ml^-1 ^acetylated BSA and 0.1 mg ml^-1 ^normal goat IgG (Sigma). This was followed by a final staining step in R-phycoerythrin streptavidin. The stained GeneChips were scanned on an Affymetrix GeneChip Scanner 3000 7G at an emission wavelength of 570 nm at a 3 pixels resolution and stored as binary CEL files. Prior to further data analysis CEL files were background corrected, quantiles normalised and median-polished summarised using the robust multi-array averaging algorithm [44] with R/Bioconductor Software [45].

### Definition of CNV genes

A chip definition file (CDF) was generated in house after mapping each probe onto the version of the 3D7 genome sequence available on 31^st ^October 2007 http://www.genedb.org, and specifying probesets consisting solely of probes mapping to individual genes and excluding all intergenic probes. For statistical purposes, only probesets with a minimum of 10 probes were retained for analysis. Log_2 _ratios were generated using median probeset hybridisation intensity signals from all hybridised strains as a comparator for individual strain probeset hybridisation intensities, the resultant values representing relative copy number on a Log_2 _scale and referred to as Log_2 _ratio hybridisation signal intensity. Cut-off values of +1/-1 represent a doubling or halving of Log_2 _hybridisation signal, probesets outside this threshold were classed as amplified or deleted respectively. This threshold limited detection of CNV genes to those >4 standard deviations from the mean, this criteria has previously been demonstrated to result in very low false positivity when detecting copy number variants [16,46]. The gene annotation used was from the 3D7 genome annotation as listed in PlasmoDB http://www.PlasmoDB.org on 31^st ^October 2007.

### Statistical analysis

Sub-telomeric regions indicated in the analysis were defined as those distal to chromosomal boundaries as previously defined [31,32], based upon homologous gene organisation between *P. falciparum *and rodent malaria parasite chromosomes. Proximity to telomeres and centromeres and possession of orthologues, transmembrane domains and signal peptides were derived from publically available datasets on http://www.PlasmoDB.org. Nucleotide diversity in 3,539 predicted genes among 5 different *P. falciparum *isolates was calculated previously [8]. Differences in distributions of values of π between groups was determined using the Kolmogorov-Smirnov test applied in the stats package in R. Average pairwise nucleotide diversity was used as a cut-off to group genes into those with values of π above or below the genome-wide average, statistically significant differences between observed and expected proportions of genes with a higher than average value of π was calculated using a Pearson's corrected chi-squared test using the on-line calculator at http://faculty.vassar.edu/lowry/odds2x2.html. Heatmaps were plotted using the Heatplus package in R/Bioconductor.

### Bioinformatic searching of the Dd2 and Hb3 genomes

Whole genome sequence data was browsed using the Broad Institute website for parasite sequence http://www.broad.mit.edu/annotation/genome/plasmodium_falciparum_spp/Regions.html. The 3D7 sequence of genes considered as 'deleted' in Hb3 or Dd2 on the basis of microarray hybridisation analysis was BLASTed against Hb3/Dd2 genomic sequence using the default settings (E-value > 1 × 10^-3^, using gapped alignments and unfiltered sequence). As a comparison to test the sensitivity of this validation approach, 500 genes that were not considered to be deleted were selected and similarly BLASTed against the Hb3 and Dd2 genome sequences (this sub-set of 500 genes had a similar distribution of probe number, gene size and AT content to the genome-wide distribution). Member of the *var*, *rifin *and *stevor *genes families were also removed prior to analysis due to the greater likelihood of misalignment in these multi-copy gene families.

## Abbreviations

CNV: copy number variation; SNP: single nucleotide polymorphism; π: nucleotide diversity; *pfsbp1*: skeleton binding protein; *rex*: ring exported protein; *clag9*: cytoadherence linked asexual protein; *pfmdr1*: multi-drug resistance protein 1; *gbph*: glycophorein binding protein homologue; *gbph2*: glycophorin binding protein homologue 2; *gbp130*: glycophorein-binding protein 130 precursor; *msp1*: merozoite surface protein 1; *pfmc-2tm*: maurer's cleft 2 transmembrane protein.

The oligonucleotide array data from this study have been submitted to ArrayExpress under accession number E-TABM-589.

## Authors' contributions

IHC, KKAT and LS cultured parasites lines, extracted genomic DNA and genotyped parasites. IHC and CKC performed microarray hybridisation and normalisation. IHC, NGE, CKC, AI and DJC analysed the data. IHC and DJC conceived and designed the experiments. IHC, NGE and DJC wrote the paper. All authors read and approved the manuscript.

## Supplementary Material

Additional file 1 **Supplementary Table 1**. A summary of the chromosome, size and product of all CNV genes detected. Click here for file

Additional file 2 **Supplementary Figure 1**. Amplified and deleted genes across all 16 isolates. Click here for file

Additional file 3 **Supplementary Table 2**. A summary of the validated deletions in the Dd2 and Hb3 genome sequences. Click here for file

Additional file 4 **Supplementary Figure 2**. Nucleotide diversity in non-variable, amplified and deleted genes. Click here for file

Additional file 5 **Supplementary Figure 3**. AT tract density in CNV and non-CNV genes. Click here for file

Additional file 6 **Supplementary Figure 4**. Correlation between hybridisation signal and SNPs in Hb3 and Dd2. Click here for file

Additional file 7 **Supplementary figure legends**. Supplementary figure legends for supplementary figures 1-4. Click here for file
