# Supplementary material for: Gene copy number variation throughout the Plasmodium falciparum genome
Source: BMC Genomics. 2009 Aug 4;10:353. doi: 10.1186/1471-2164-10-353 (PMC2732925; doi:10.1186/1471-2164-10-353)
Supplement: Additional file 1 — Supplementary Table 1. A summary of the chromosome, size and product of all CNV genes detected. [file 1471-2164-10-353-S1.doc]

| **Supplementary Table 1.** | |  |  |  |  |  |
| --- | --- | --- | --- | --- | --- | --- |
|  |  |  |  |  |  |  |
| **CNV genes present in a single strain are highlghted in grey** | | | | |  |  |
|  |  |  |  |  |  |  |
| **Probeset** | **Amplifications** | **Deletions** | **total CNVs** | **Chr** | **Size (bp)** | **Gene product** |
| PFA0025c | 1 | 2 | 3 | 1 | 111 | erythrocyte membrane protein 1 (PfEMP1) pseudogene |
| PFA0035c | 1 | 0 | 1 | 1 | 203 | hypothetical protein, conserved in P. falciparum |
| PFA0095c | 1 | 0 | 1 | 1 | 1178 | rifin |
| PFA0120c | 0 | 1 | 1 | 1 | 1151 | hydrolase, putative |
| PFA0130c | 0 | 1 | 1 | 1 | 2373 | Serine/Threonine protein kinase, FIKK family |
| PFA0365c | 1 | 0 | 1 | 1 | 653 | hypothetical protein, conserved in P. falciparum |
| PFA0420w | 1 | 0 | 1 | 1 | 539 | hypothetical protein, conserved |
| PFA0645c | 3 | 0 | 3 | 1 | 245 | hypothetical protein |
| PFA0680c | 1 | 0 | 1 | 1 | 783 | Plasmodium falcioparum maurer's cleft 2 transmembrane domain protein 1.2, PfMC-2TM_1.2 |
| PFA0730c | 2 | 0 | 2 | 1 |  | Gene model removed since reannotation of *P. falciparum* genome |
| PFA0735w | 0 | 1 | 1 | 1 | 1003 | hypothetical protein, conserved in P.falciparum |
| PFA0755w | 1 | 0 | 1 | 1 | 432 | erythrocyte membrane protein 1 (PfEMP1) pseudogene |
| PFA0760w | 1 | 0 | 1 | 1 | 1369 | rifin |
|  |  |  |  |  |  |  |
|  |  |  |  |  |  |  |
| PFB0045c | 5 | 3 | 8 | 2 | 1282 | erythrocyte membrane protein 1 (PfEMP1), truncated |
| PFB0070w | 1 | 3 | 4 | 2 | 683 | hypothetical protein |
| PFB0080c | 0 | 3 | 3 | 2 | 1557 | hypothetical protein |
| PFB0085c | 0 | 4 | 4 | 2 | 2874 | DnaJ protein, putative |
| PFB0090c | 0 | 2 | 2 | 2 | 1801 | DnaJ protein, putative |
| PFB0105c | 0 | 2 | 2 | 2 | 1016 | hypothetical protein |
| PFB0951w | 0 | 1 | 1 | 2 | 538 | hypothetical protein, pseudogene |
| PFB0953w | 0 | 1 | 1 | 2 | 693 | hypothetical protein |
| PFB0954c | 0 | 1 | 1 | 2 | 610 | hypothetical protein, conserved in P falciparum |
| PFB0955w | 0 | 1 | 1 | 2 | 1149 | stevor, degenerate, putative |
| PFB0965c | 0 | 1 | 1 | 2 | 410 | hypothetical protein |
| PFB0970c | 0 | 2 | 2 | 2 | 1616 | hypothetical protein |
| PFB0973c | 0 | 1 | 1 | 2 | 194 | hypothetical protein |
| PFB0974c | 2 | 2 | 4 | 2 | 194 | erythrocyte membrane protein 1 (PfEMP1), truncated, degenerate |
| PFB0985c | 2 | 0 | 2 | 2 | 783 | Plasmodium falciparum Maurer's Cleft 2 transmembrane domain protein 2.2, PfMC-2TM_2.2 |
| PFB0995w | 0 | 2 | 2 | 2 | 773 | hypothetical protein |
| PFB1000w | 1 | 0 | 1 | 2 | 887 | rifin |
| PFB1005w | 1 | 0 | 1 | 2 | 1085 | rifin |
| PFB1025w | 3 | 0 | 3 | 2 | 257 | erythrocyte membrane protein 1 (PfEMP1), truncated, degenerate |
| PFB1030w | 2 | 0 | 2 | 2 | 353 | hypothetical protein |
| PFB1045w | 1 | 0 | 1 | 2 | 1704 | erythrocyte membrane protein 1 (PfEMP1), truncated |
| PFB1060w | 0 | 3 | 3 | 2 |  | Gene model removed since reannotation of *P. falciparum* genome |
| PFB1065c | 3 | 0 | 3 | 2 |  | Gene model removed since reannotation of *P. falciparum* genome |
|  |  |  |  |  |  |  |
|  |  |  |  |  |  |  |
| PFC0002c | 0 | 2 | 2 | 3 | 2351 | hypothetical protein, conserved in P. falciparum |
| PFC0015c | 2 | 1 | 3 | 3 | 1380 | VAR C, pseudogene |
| PFC0040w | 3 | 0 | 3 | 3 | 1211 | rifin |
| PFC0045w | 0 | 1 | 1 | 3 | 904 | rifin-like protein |
| PFC0400w | 0 | 3 | 3 | 3 | 338 | 60S Acidic ribosomal protein P2 |
| PFC0486c | 0 | 1 | 1 | 3 | 245 | hypothetical protein, conserved |
| PFC0845c | 0 | 1 | 1 | 3 | 323 | ubiquitin--protein ligase, putative |
| PFC1070c | 1 | 0 | 1 | 3 | 874 | VARC pseudogene |
| PFC1080c | 0 | 1 | 1 | 3 | 789 | Plasmodium falciparum Maurer's Cleft 2 transmembrane domain protein 3, PfMC-2TM_3 |
| PFC1105w | 1 | 0 | 1 | 3 | 1010 | stevor (3D7-stevorT3-2) |
| PFC1125w | 0 | 3 | 3 | 3 | 4117 | hypothetical protein, conserved in P. falciparum |
|  |  |  |  |  |  |  |
|  |  |  |  |  |  |  |
| PFD0040c | 1 | 0 | 1 | 4 | 1152 | rifin |
| PFD0060w | 1 | 0 | 1 | 4 | 1211 | rifin |
| PFD0134c | 0 | 1 | 1 | 4 | 503 | RIF pseudogene, RIFIN pseudogene |
| PFD0315c | 0 | 2 | 2 | 4 | 442 | hypothetical protein |
| PFD0365c | 0 | 1 | 1 | 4 | 308 | hypothetical protein |
| PFD0595w | 1 | 0 | 1 | 4 | 2337 | hypothetical protein, conserved |
| PFD1000c | 6 | 4 | 10 | 4 | 7604 | erythrocyte membrane protein 1 (PfEMP1) |
| PFD1007w | 0 | 1 | 1 | 4 | 215 | hypothetical protein |
| PFD1190c | 1 | 0 | 1 | 4 | 368 | hypothetical protein |
| PFD1200c | 2 | 0 | 2 | 4 | 761 | hypothetical protein, conserved in P.falciparum |
| PFD1250w | 1 | 2 | 3 | 4 | 2993 | hypothetical protein, conserved in P falciparum |
|  |  |  |  |  |  |  |
|  |  |  |  |  |  |  |
| PFE0035c | 0 | 1 | 1 | 5 | 274 | RIF pseudogene, RIFIN pseudogene |
| PFE0065w | 0 | 2 | 2 | 5 | 1183 | skeleton binding protein1, PfSBP1 |
| PFE0070w | 0 | 3 | 3 | 5 | 5333 | interspersed repeat antigen, putative |
| PFE1095w | 1 | 0 | 1 | 5 | 6070 | hypothetical protein, conserved |
| PFE1100w | 2 | 0 | 2 | 5 | 922 | hypothetical protein, conserved |
| PFE1105c | 2 | 0 | 2 | 5 | 2291 | hypothetical protein, conserved |
| PFE1110w | 1 | 0 | 1 | 5 | 759 | hypothetical protein, conserved |
| PFE1115c | 2 | 0 | 2 | 5 | 2231 | s-adenosylmethionine-dependent methyltransferase, putative |
| PFE1120w | 1 | 0 | 1 | 5 | 28478 | hypothetical protein, conserved |
| PFE1125w | 2 | 0 | 2 | 5 | 722 | 50S ribosomal subunit protein L17, putative |
| PFE1130w | 2 | 0 | 2 | 5 | 1451 | hypothetical protein, conserved |
| PFE1135w | 2 | 0 | 2 | 5 | 1106 | hypothetical protein, conserved |
| PFE1140c | 1 | 0 | 1 | 5 | 747 | G10 protein, putative |
| PFE1145w | 1 | 0 | 1 | 5 | 4658 | hypothetical protein, conserved |
| PFE1150w | 2 | 0 | 2 | 5 | 4259 | multidrug resistance protein |
| PFE1155c | 1 | 0 | 1 | 5 | 1817 | mitochondrial processing peptidase alpha subunit, putative |
| PFE1160w | 1 | 0 | 1 | 5 | 3614 | hypothetical protein, conserved |
| PFE1230c | 1 | 0 | 1 | 5 | 317 | hypothetical protein, conserved |
| PFE1590w | 0 | 1 | 1 | 5 | 545 | early transcribed membrane protein 5, ETRAMP5 |
| PFE1625c | 1 | 0 | 1 | 5 | 287 | erythrocyte membrane protein 1 (PfEMP1) pseudogene |
|  |  |  |  |  |  |  |
|  |  |  |  |  |  |  |
| PFF0005c | 1 | 0 | 1 | 6 | 779 | erythrocyte membrane protein 1 (PfEMP1) pseudogene |
| PFF0080c | 1 | 0 | 1 | 6 | 860 | hypothetical protein, conserved |
| PFF0510w | 1 | 1 | 2 | 6 | 410 | histone H3, putative |
| PFF0860c | 1 | 1 | 2 | 6 | 398 | histone h2a |
| PFF1505w | 1 | 0 | 1 | 6 | 857 | hypothetical protein, conserved |
| PFF1555w | 1 | 0 | 1 | 6 | 1327 | rifin |
| PFF1560c | 3 | 0 | 3 | 6 | 1199 | rifin |
| PFF1585w | 4 | 0 | 4 | 6 | 1192 | rifin pseudogene |
|  |  |  |  |  |  |  |
|  |  |  |  |  |  |  |
| MAL7P1.183 | 2 | 0 | 2 | 7 | 1284 | erythrocyte membrane protein 1 (PfEMP1) pseudogene |
| MAL7P1.186 | 1 | 0 | 1 | 7 | 1796 | VAR-like erythrocyte membrane protein 1 |
| MAL7P1.212 | 3 | 1 | 4 | 7 | 7655 | erythrocyte membrane protein 1 (PfEMP1) |
| MAL7P1.320 | 1 | 1 | 2 | 7 | 278 | Ribosomal protein, L37e, putative |
| MAL7P1.5 | 1 | 0 | 1 | 7 | 811 | Plasmodium falciparum Maurer's Cleft 2 transmembrane domain protein, PfMC-2TM_7.1 |
| MAL7P1.6 | 0 | 1 | 1 | 7 | 892 | hypothetical protein, conserved in P.falciparum |
| MAL7P1.75 | 1 | 0 | 1 | 7 | 514 | mitochondrial ATP synthase F1, epsilon subunit, putative |
| PF07_0002 | 2 | 4 | 6 | 7 | 848 | hypothetical protein, conserved in P. falciparum |
| PF07_0005 | 0 | 1 | 1 | 7 | 1274 | lysophospholipases-like protein, putative |
| PF07_0027 | 0 | 1 | 1 | 7 | 209 | DNA-directed RNA polymerase 2 8.2 kDa polypeptide, putative |
| PF07_0093 | 0 | 1 | 1 | 7 | 416 | hypothetical protein, conserved |
| PF07_0138 | 1 | 0 | 1 | 7 | 1249 | rifin |
|  |  |  |  |  |  |  |
|  |  |  |  |  |  |  |
| MAL8P1.147 | 3 | 2 | 5 | 8 | 311 | hypothetical protein |
| MAL8P1.163 | 2 | 0 | 2 | 8 | 997 | hypothetical protein, conserved in P.falciparum |
| MAL8P1.310 | 1 | 0 | 1 | 8 | 399 | putative senescence-associated protein |
| MAL8P1.330 | 0 | 3 | 3 | 8 | 620 | hypothetical protein, conserved in P.falciparum |
| MAL8P1.335 | 0 | 2 | 2 | 8 | 2906 | hypothetical protein, conserved in P. falciparum |
| MAL8P1.87 | 1 | 0 | 1 | 8 | 215 | hypothetical protein |
| MAL8P1.90 | 0 | 2 | 2 | 8 | 125 | hypothetical protein |
| PF08_0025 | 1 | 0 | 1 | 8 | 257 | hypothetical protein, conserved |
| PF08_0057 | 1 | 0 | 1 | 8 | 301 | hypothetical protein, conserved |
| PF08_0074 | 0 | 2 | 2 | 8 | 746 | DNA/RNA-binding protein Alba, putative |
| PF08_0119 | 1 | 0 | 1 | 8 | 281 | hypothetical protein, conserved |
|  |  |  |  |  |  |  |
|  |  |  |  |  |  |  |
| PFI0025c | 1 | 0 | 1 | 9 | 1136 | rifin |
| PFI0060c | 2 | 2 | 4 | 9 | 212 | hypothetical protein, conserved in P.falciparum |
| PFI0620w | 1 | 0 | 1 | 9 | 257 | hypothetical protein, conserved |
| PFI0790w | 1 | 0 | 1 | 9 | 657 | thioredoxin, putative |
| PFI1405c | 0 | 1 | 1 | 9 | 341 | hypothetical protein, conserved |
| PFI1720w | 0 | 2 | 2 | 9 | 1830 | Gametocyte-implicated protein (Fragment) |
| PFI1725w | 0 | 3 | 3 | 9 | 809 | hypothetical protein, conserved |
| PFI1730w | 0 | 1 | 1 | 9 | 5914 | cytoadherence linked asexual protein 9(CLAG9) |
| PFI1740c | 0 | 5 | 5 | 9 | 548 | hypothetical protein |
| PFI1745c | 1 | 7 | 8 | 9 | 539 | hypothetical protein, conserved |
| PFI1755c | 0 | 2 | 2 | 9 | 1294 | hypothetical protein, conserved |
| PFI1760w | 2 | 7 | 9 | 9 | 877 | hypothetical protein, conserved |
| PFI1780w | 0 | 3 | 3 | 9 | 1470 | hypothetical protein |
| PFI1785w | 7 | 0 | 7 | 9 | 1259 | hypothetical protein, conserved in P.falciparum |
| PFI1795c | 3 | 0 | 3 | 9 | 871 | hypothetical protein |
|  |  |  |  |  |  |  |
|  |  |  |  |  |  |  |
| PF10_0007 | 0 | 1 | 1 | 10 | 227 | hypothetical protein |
| PF10_0008 | 0 | 3 | 3 | 10 | 384 | hypothetical protein |
| PF10_0074 | 0 | 1 | 1 | 10 | 329 | hypothetical protein |
| PF10_0353 | 1 | 1 | 2 | 10 | 86 | hypothetical protein |
| PF10_0383 | 5 | 4 | 9 | 10 | 1013 | hypothetical protein, conserved |
| PF10_0388 | 1 | 0 | 1 | 10 | 515 | hypothetical protein |
| PF10_0390 | 1 | 0 | 1 | 10 | 789 | Plasmodium falciparum Maurer's Cleft 2 transmembrane domain protein 10, PfMC-2TM_10 |
| PF10_0394 | 1 | 0 | 1 | 10 | 1054 | rifin |
| PF10_0398 | 1 | 0 | 1 | 10 | 1266 | rifin |
| PF10_0399 | 1 | 0 | 1 | 10 | 1158 | rifin |
|  |  |  |  |  |  |  |
|  |  |  |  |  |  |  |
| PF11_0002 | 0 | 4 | 4 | 11 |  | Gene model removed since reannotation of *P. falciparum* genome |
| PF11_0003 | 1 | 0 | 1 | 11 |  | Gene model removed since reannotation of *P. falciparum* genome |
| PF11_0004 | 1 | 2 | 3 | 11 |  | Gene model removed since reannotation of *P. falciparum* genome |
| PF11_0005 | 0 | 1 | 1 | 11 |  | Gene model removed since reannotation of *P. falciparum* genome |
| PF11_0006 | 0 | 2 | 2 | 11 |  | Gene model removed since reannotation of *P. falciparum* genome |
| PF11_0021 | 1 | 0 | 1 | 11 | 1198 | rifin |
| PF11_0025 | 5 | 6 | 11 | 11 | 789 | Plasmodium falciparum Maurer's Cleft 2 transmembrane domain protein 11.2 |
| PF11_0033 | 5 | 4 | 9 | 11 | 2645 | hypothetical protein |
| PF11_0040 | 0 | 2 | 2 | 11 | 284 | early transcribed membrane protein 11.2, etramp11.2 |
| PF11_0061 | 0 | 3 | 3 | 11 | 311 | histone H4, putative |
| PF11_0106 | 0 | 1 | 1 | 11 | 392 | 60S Ribosomal protein L36, putative |
| PF11_0136 | 0 | 1 | 1 | 11 | 320 | hypothetical protein |
| PF11_0340 | 1 | 1 | 2 | 11 | 125 | hypothetical protein |
| PF11_0423 | 1 | 0 | 1 | 11 | 471 | hypothetical protein |
| PF11_0454 | 1 | 0 | 1 | 11 | 497 | Ribosomal protein, 40S subunit, putative |
| PF11_0475 | 1 | 0 | 1 | 11 | 203 | hypothetical protein |
| PF11_0504 | 1 | 0 | 1 | 11 | 908 | hypothetical protein |
| PF11_0514 | 1 | 1 | 2 | 11 | 257 | hypothetical protein |
| PF11_0525 | 0 | 1 | 1 | 11 | 305 | hypothetical protein |
|  |  |  |  |  |  |  |
|  |  |  |  |  |  |  |
| PFL0957w | 1 | 0 | 1 | 12 | 366 | hypothetical protein |
| PFL1135c | 1 | 0 | 1 | 12 | 4860 | hypothetical protein, conserved |
| PFL1140w | 1 | 0 | 1 | 12 | 1093 | hypothetical protein, conserved |
| PFL1145w | 2 | 0 | 2 | 12 | 926 | hypothetical protein, conserved |
| PFL1150c | 2 | 0 | 2 | 12 | 683 | ribosomal protein L24, putative |
| PFL2555w | 0 | 1 | 1 | 12 | 1007 | hypothetical protein, conserved in P. falciparum |
| PFL2560c | 0 | 1 | 1 | 12 | 326 | hypothetical protein, conserved in P. falciparum |
| PFL2565w | 0 | 2 | 2 | 12 | 758 | hypothetical protein, consevred in P. falciparum |
| PFL2575c | 1 | 3 | 4 | 12 | 704 | hypothetical protein, conserved in P. falciparum |
| PFL2590w | 6 | 2 | 8 | 12 | 965 | hypothetical protein, onserved in P. falciparum |
| PFL2595w | 1 | 0 | 1 | 12 | 631 | hypothetical protein, conserved in P. falciparum |
|  |  |  |  |  |  |  |
|  |  |  |  |  |  |  |
| MAL13P1.490 | 0 | 1 | 1 | 13 | 1083 | StevoR |
| MAL13P1.510 | 4 | 0 | 4 | 13 | 1304 | erythrocyte membrane protein 1 (PfEMP1), pseudogene |
| MAL13P1.58 | 0 | 3 | 3 | 13 | 790 | hypothetical protein, conserved in P. falciparum |
| MAL13P1.61 | 0 | 2 | 2 | 13 | 973 | hypothetical protein, conserved in P. falciparum |
| MAL13P1.8 | 2 | 0 | 2 | 13 | 1268 | RIF pseudogene |
| PF13_0010 | 0 | 3 | 3 | 13 | 1314 | Gbph2 |
| PF13_0076 | 0 | 1 | 1 | 13 | 1023 | hypothetical protein, conserved |
| PF13_0224 | 0 | 1 | 1 | 13 | 670 | 60S ribosomal subunit protein L18, putative |
| PF13_0305 | 0 | 1 | 1 | 13 | 1331 | elongation factor 1 alpha |
| PF13_0347 | 1 | 0 | 1 | 13 | 275 | hypothetical protein, conserved |
|  |  |  |  |  |  |  |
|  |  |  |  |  |  |  |
| PF14_0010 | 0 | 3 | 3 | 14 | 1080 | glycophorin binding protein-related antigen |
| PF14_0013 | 0 | 1 | 1 | 14 | 1861 | hypothetical protein |
| PF14_0040 | 0 | 1 | 1 | 14 | 608 | hypothetical protein |
| PF14_0118 | 0 | 2 | 2 | 14 | 292 | hypothetical protein |
| PF14_0194 | 0 | 1 | 1 | 14 | 1869 | spliceosome-associated protein, putative |
| PF14_0340 | 1 | 0 | 1 | 14 | 854 | hypothetical protein |
| PF14_0590 | 0 | 2 | 2 | 14 | 365 | hypothetical protein |
| PF14_0592 | 2 | 0 | 2 | 14 | 221 | hypothetical protein |
| PF14_0734 | 0 | 1 | 1 | 14 | 851 | protein kianse, FIKK family |
| PF14_0742 | 0 | 1 | 1 | 14 | 672 | hypothetical protein |
| PF14_0762 | 4 | 0 | 4 | 14 | 706 | hypothetical protein |
| PF14_0763 | 4 | 4 | 8 | 14 | 982 | hypothetical protein |
