# Supplementary figures and images for: Gene copy number variation throughout the Plasmodium falciparum genome
Source: BMC Genomics. 2009 Aug 4;10:353. doi: 10.1186/1471-2164-10-353 (PMC2732925; doi:10.1186/1471-2164-10-353)

## Slide 1
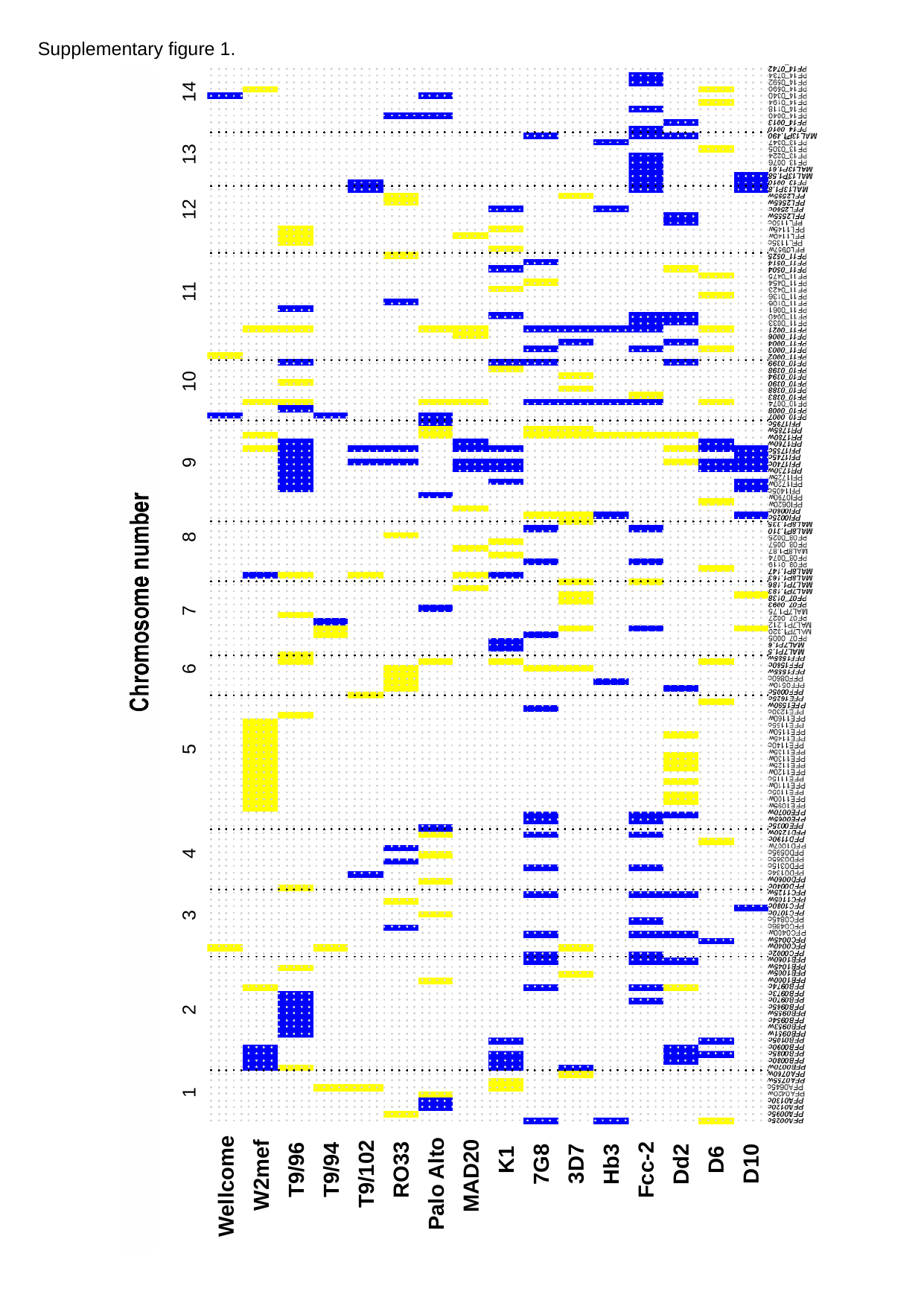

Supplementary figure 1.
14
13
12
11
10
9
8
7
6
5
4
3
2
1
K1
D6
3D7
D10
7G8
Hb3
Dd2
T9/96
T9/94
RO33
Fcc-2
T9/102
W2mef
MAD20
Palo Alto
Wellcome

Supplement: Additional file 2 — Supplementary Figure 1. Amplified and deleted genes across all 16 isolates. [file 1471-2164-10-353-S2.ppt]

## Slide 1
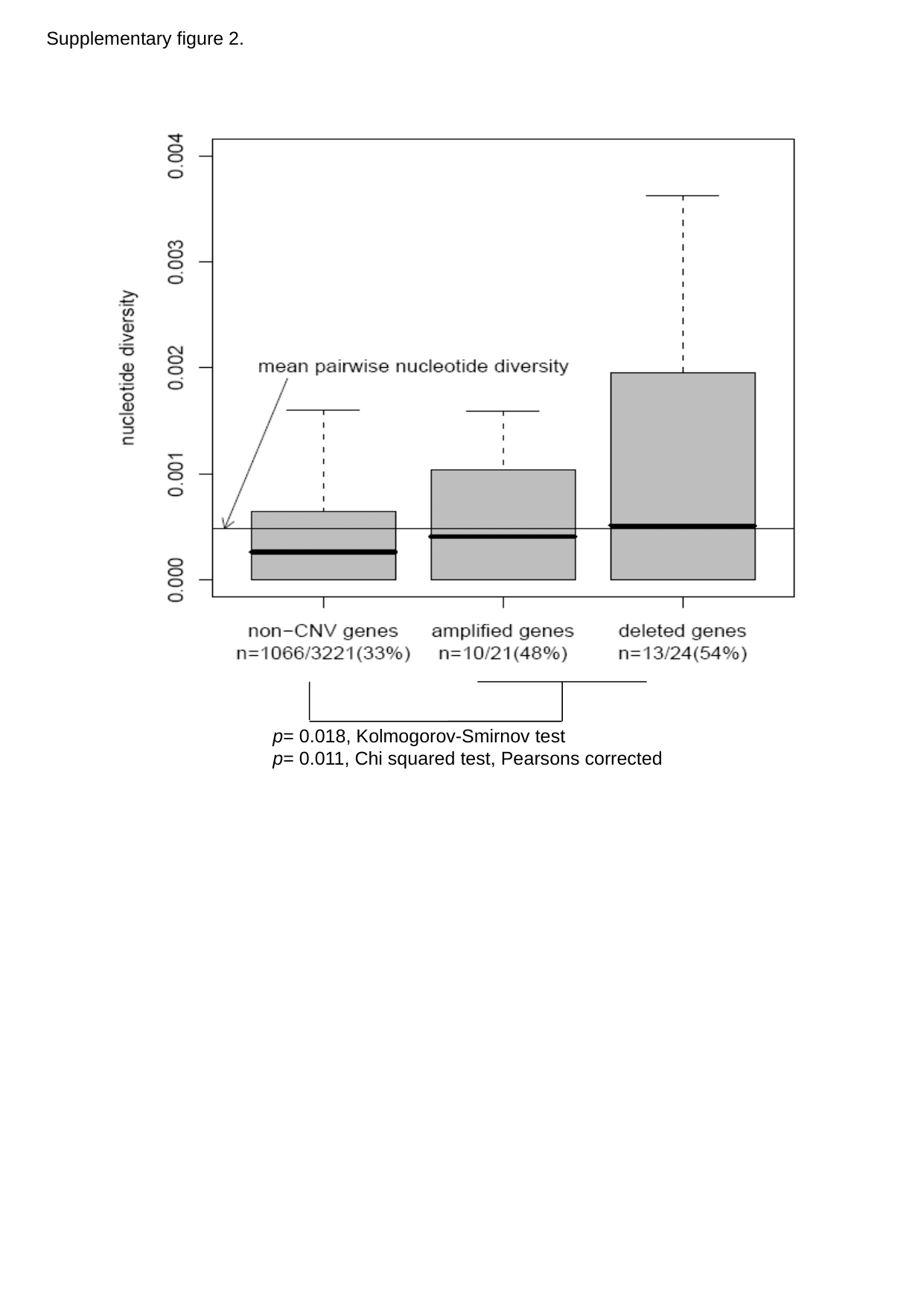

Supplementary figure 2.
p= 0.018, Kolmogorov-Smirnov test
p= 0.011, Chi squared test, Pearsons corrected

Supplement: Additional file 4 — Supplementary Figure 2. Nucleotide diversity in non-variable, amplified and deleted genes. [file 1471-2164-10-353-S4.ppt]

## Slide 1
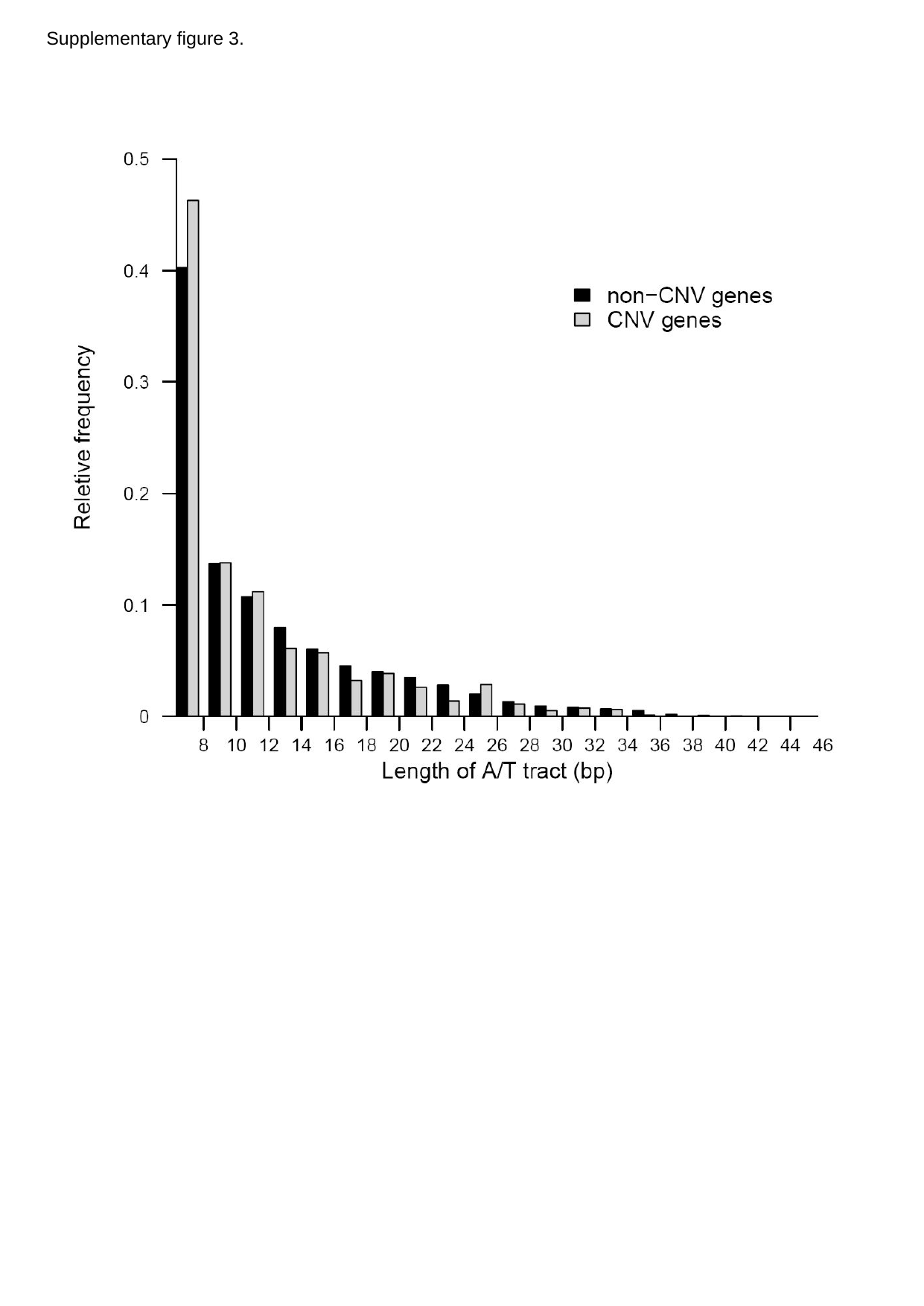

Supplementary figure 3.

Supplement: Additional file 5 — Supplementary Figure 3. AT tract density in CNV and non-CNV genes. [file 1471-2164-10-353-S5.ppt]
