# Supplementary material for: Gene copy number variation throughout the Plasmodium falciparum genome
Source: BMC Genomics. 2009 Aug 4;10:353. doi: 10.1186/1471-2164-10-353 (PMC2732925; doi:10.1186/1471-2164-10-353)
Supplement: Additional file 3 — Supplementary Table 2. A summary of the validated deletions in the Dd2 and Hb3 genome sequences. [file 1471-2164-10-353-S3.doc]

**Supplementary Table 2. Validation of deletions in the Hb3 and Dd2 genomes**

| **Gene** | **Strain** | **Size** | **Probe number** | **Log_2_ signal ratio** | **Notes*** |
| --- | --- | --- | --- | --- | --- |
| **PFA0025c** | **Hb3** | **112bp** | **38** | **-2.2** | **no BLAST hits map to PFA0025c** |
| PFF0860c | Hb3 | 399bp | 24 | -1.15 | 14bp deletion in Hb3 overlaps 17 of 38 (44.73%) probes |
| PFI0060c | Hb3 | 213bp | 12 | -1.72 | Polymorphisms occuring in 12 of 12 probes (100%) |
| **PF10_0383** | **Hb3** | **1014bp** | **29** | **-1.69** | **no BLAST hits map to PF10_0383** |
| **PF11_0033** | **Hb3** | **2646bp** | **14** | **-1.63** | **no BLAST hits map to PF11_0033** |
| **PFL2565w** | **Hb3** | **543bp** | **61** | **-3.57** | **357bp truncation** |
| **MAL13P1.490** | **Hb3** | **726bp** | **59** | **-1.37** | **no BLAST hits map to MAL13P1.490** |
|  |  |  |  |  |  |
| **PFB0080c** | **Dd2** | **1458bp** | **131** | **-1.09** | **no BLAST hits map to PFB0080c** |
| **PFB0085c** | **Dd2** | **2703bp** | **241** | **-1.30** | **no BLAST hits map to PFB0085c** |
| **PFB0090c** | **Dd2** | **1206bp** | **136** | **-1.23** | **no BLAST hits map to PFB0090c** |
| **PFC0400w** | **Dd2** | **339bp** | **28** | **-1.29** | **no BLAST hits map to PFC0400w** |
| **PFC1125w** | **Dd2** | **4118bp** | **137** | **-1.12** | **no BLAST hits map to PFC1125w** |
| **PFE0065w** | **Dd2** | **1184bp** | **92** | **-0.95** | **740bp truncation of PFE0065w** |
| PFE0070w | Dd2 | 5334bp | 214 | -1.15 | Extensive rearrangements of interspersed repeat sequences |
| **PFF0510w** | **Dd2** | **411bp** | **39** | **-1.08** | **no BLAST hits map to PFF0510w** |
| **PF11_0040** | **Dd2** | **285bp** | **26** | **-1.00** | **no BLAST hits map to PF11_0040** |
| PF11_0061 | Dd2 | 312bp | 30 | -1.01 | 74bp truncation overlapping 10 of 30 probes |
| **PFL2555w** | **Dd2** | **1008bp** | **84** | **-1.60** | **no BLAST hits map to PFL2555w** |
| **PFL2560c** | **Dd2** | **327bp** | **28** | **-2.00** | **no BLAST hits map to PFL2560c** |
| **PF14_0010** | **Dd2** | **1081bp** | **234** | **-1.42** | **886bp truncation** |
| PF14_0040 | Dd2 | 609bp | 55 | -1.09 | polymorphism ocurring in 15/55 (27%) probes |

| NB BLAST results from blastn using 3D7 sequence against Hb3/Dd2 genome sequence |
| --- |
| *Genes in **bold** show deletion/truncations covering >50% of genomic sequence in the query strain |
