# Supplementary material for: Gene copy number variation throughout the Plasmodium falciparum genome
Source: BMC Genomics. 2009 Aug 4;10:353. doi: 10.1186/1471-2164-10-353 (PMC2732925; doi:10.1186/1471-2164-10-353)
Supplement: Additional file 6 — Supplementary Figure 4. Correlation between hybridisation signal and SNPs in Hb3 and Dd2. [file 1471-2164-10-353-S6.ppt]

## Slide 1
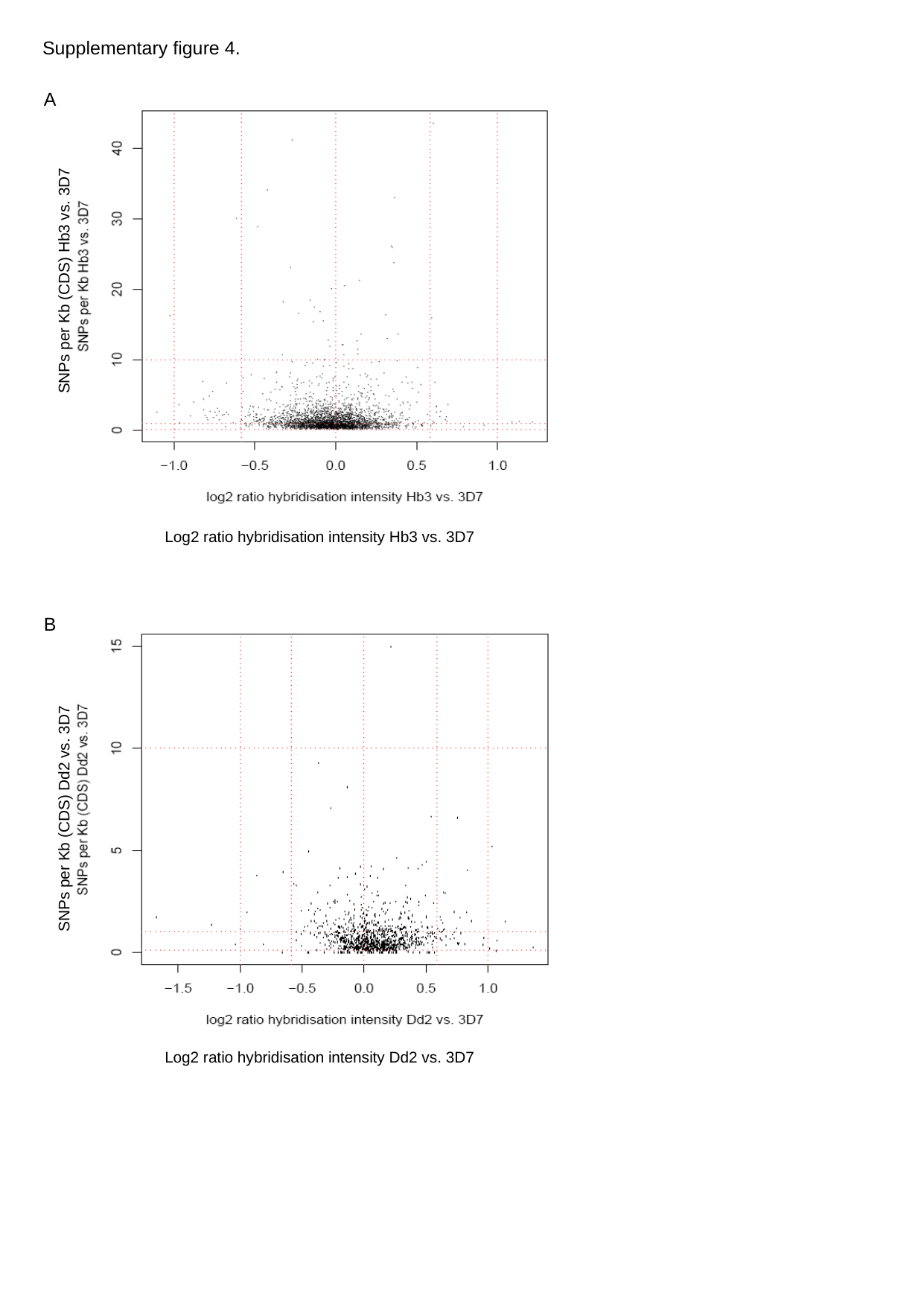

Supplementary figure 4.
A
SNPs per Kb (CDS) Hb3 vs. 3D7
Log2 ratio hybridisation intensity Hb3 vs. 3D7
B
SNPs per Kb (CDS) Dd2 vs. 3D7
Log2 ratio hybridisation intensity Dd2 vs. 3D7
