# Supplementary material for: Gene copy number variation throughout the Plasmodium falciparum genome
Source: BMC Genomics. 2009 Aug 4;10:353. doi: 10.1186/1471-2164-10-353 (PMC2732925; doi:10.1186/1471-2164-10-353)
Supplement: Additional file 7 — Supplementary figure legends. Supplementary figure legends for supplementary figures 1-4. [file 1471-2164-10-353-S7.doc]

**Supplementary Figure 1.** Amplified (yellow) and deleted (blue) genes across all 16 hybridisations. Genes are ordered by physical order along the chromosome with sub-telomeric genes shown in bold and italics. Intervening non-CNV genes were not plotted.

**Supplementary Figure 2**. Nucleotide diversity in non-variable, amplified and deleted genes. The distribution of values of π for CNV showed a significant divergence from those for non-CNV genes. A chi squared test also revealed that the proportion of genes with values for π in excess of the mean pairwise nucleotide diversity was significantly increased in CNV genes compared to non-CNV genes.

**Supplementary Figure 3.** Distribution of homopolymeric A/T tract lengths within and flanking (surrounding 1 kb) of gene coding sequences, compared between CNV genes (n = 186) and non-CNV genes (n = 5122).

**Supplementary Figure 4.** Correlation of nucleotide polymorphism in genes with probeset (gene) level hybridisation signals. Hb3 (A) and Dd2 (B) parasite clones showed no relationship between hybridisation signal and numbers of SNPs per kb of coding sequence, suggesting probeset level analysis, such as CNV detection, on the PFSANGER microarray is insensitive to effects of the small numbers of SNPs in most genes.
